# Methamphetamine alters microglial immune function through P2X7R signaling

**DOI:** 10.1186/s12974-016-0553-3

**Published:** 2016-04-26

**Authors:** Nicole C. Fernandes, Uma Sriram, Larisa Gofman, Jonathan M. Cenna, Servio H. Ramirez, Raghava Potula

**Affiliations:** Department of Pathology and Laboratory Medicine, Lewis Katz School of Medicine at Temple University, MERB 845A, 3500 N. Broad Street, Philadelphia, 19140 PA USA; Center for Substance Abuse Research, Lewis Katz School of Medicine, Philadelphia, PA USA

**Keywords:** Microglia, Methamphetamine, Purinergic receptor X7

## Abstract

**Background:**

Purinoceptors have emerged as mediators of chronic inflammation and neurodegenerative processes. The ionotropic purinoceptor P2X7 (P2X7R) is known to modulate proinflammatory signaling and integrate neuronal-glial circuits. Evidence of P2X7R involvement in neurodegeneration, chronic pain, and chronic inflammation suggests that purinergic signaling plays a major role in microglial activation during neuroinflammation. In this study, we investigated the effects of methamphetamine (METH) on microglial P2X7R.

**Methods:**

ESdMs were used to evaluate changes in METH-induced P2X7R gene expression via Taqman PCR and protein expression via western blot analysis. Migration and phagocytosis assays were used to evaluate functional changes in ESdMs in response to METH treatment. METH-induced proinflammatory cytokine production following siRNA silencing of P2X7R in ESdMs measured P2X7R-dependent functional changes. In vivo expression of P2X7R and tyrosine hydroxylase (TH) was visualized in an escalating METH dose mouse model via immunohistochemical analysis.

**Results:**

Stimulation of ESdMs with METH for 48 h significantly increased P2X7R mRNA (*p < 0.0336) and protein expression (*p < 0.022). Further analysis of P2X7R protein in cellular fractionations revealed increases in membrane P2X7R (*p < 0.05) but decreased cytoplasmic expression after 48 h METH treatment, suggesting protein mobilization from the cytoplasm to the membrane which occurs upon microglial stimulation with METH. Forty-eight hour METH treatment increased microglial migration towards Fractalkine (CX3CL1) compared to control (****p < 0.0001). Migration toward CX3CL1 was confirmed to be P2X7R-dependent through the use of A 438079, a P2X7R-competitive antagonist, which reversed the METH effects (****p < 0.0001). Similarly, 48 h METH treatment increased microglial phagocytosis compared to control (****p < 0.0001), and pretreatment of P2X7R antagonist reduced METH-induced phagocytosis (****p < 0.0001). Silencing the microglial P2X7R decreased TNF-α (*p < 0.0363) and IL-10 production after 48 h of METH treatment. Additionally, our studies demonstrate increased P2X7R and decreased TH expression in the striata of escalating dose METH animal model compared to controls.

**Conclusions:**

This study sheds new light on the functional role of P2X7R in the regulation of microglial effector functions during substance abuse. Our findings suggest that P2X7R plays an important role in METH-induced microglial activation responses. P2X7R antagonists may thus constitute a novel target of therapeutic utility in neuroinflammatory conditions by regulating pathologically activated glial cells in stimulant abuse.

**Electronic supplementary material:**

The online version of this article (doi:10.1186/s12974-016-0553-3) contains supplementary material, which is available to authorized users.

## Background

Microglia, the resident macrophages of the CNS, are the driving force behind the neuroinflammatory response to CNS injury. As the immune sentinels and first responders, microglia constantly survey the environment by extending and retracting their processes [1, 2]. Microglial activation, also known as microgliosis, may occur as a consequence of a single stimulus or multiple stimuli. During microgliosis, microglia transition from a surveillant, ramified phenotype to an amoeboid, motile phenotype with migratory and phagocytic capacities [1, 3, 4]. Microgliosis is also associated with the generation of neuroinflammatory mediators and cellular expansion [1, 3]. Acute microgliosis is transient and is usually resolved within 1 month after the initial activation [5, 6]. However, chronic microgliosis is the result of hyperactive microglia creating a continuous, self-propagating feedback loop that leads to sustained neuroinflammation. Sustained inflammation further promotes the generation and accumulation of neurotoxic inflammatory mediators that contribute to neural damage in neurodegenerative diseases and psychostimulant drug abuse [1, 3].

Although the molecular mechanisms of microglial activation remain unknown, the ATP-sensitive P2 purinoceptors are implicated as the primary factor in initiating microgliosis [2]. In particular, the ligand-gated P2X7 receptor (P2X7R) is known to contribute to inflammatory responses and is implicated in several neurodegenerative diseases [2, 7, 8]. P2X7R activation results in the release of proinflammatory mediators, such as IL-1β, in response to injury [2]. The concurrent increases in the expression of P2X7R and activation of microglia suggest that P2X7R-induced microglial activation may contribute to the microglial migration, phagocytosis, and cytokine release during neuroinflammation [2, 8, 9]. Methamphetamine (METH) exposure is known to promote microglial activation and production of proinflammatory cytokines and chemokines [10]. Moreover, METH-induced microgliosis appears to precede METH-induced neurotoxicity [11–13]. While the potent effects of METH are known to initiate and promote neurotoxicity, the direct effect of METH on microglia is less understood [14, 15]. Moreover, microgliosis occurs within minutes and may be sustained for up to several weeks in vivo [16]. Here, we demonstrate that METH alters microglial effector functions through P2X7R. We also demonstrate that chronic exposure of METH in vivo results in exacerbated microglial P2X7R expression, indicating a role for this receptor in regulating immune cell inflammatory responses to METH. We suggest that P2X7R plays an important role in the underlying mechanism of METH-induced microglial activation.

## Methods

### METH administration in vivo

Male C57BL/6 mice 6 weeks of age were purchased from Jackson Labs, housed in specific pathogen-free conditions, and given unlimited access to food and water. Protocols for the use of animals were in accordance with the guidelines of and were approved by the Institutional Animal Care and Use Committees of Temple University, which is an American Association for the Accreditation of Laboratory Animal Care accredited facility.

Methamphetamine hydrochloride was purchased from Sigma-Aldrich (St. Louis, MO). Mice were weight-matched and randomly divided into saline or METH groups. Mice in the METH group were subcutaneously administered a gradual escalating METH dose twice a day from 0.675–10 mg/kg over 6 days, with total doses for each day represented in Fig. 5a. Beginning on day 7, mice were subcutaneously administered a single dose of 10 mg/kg/day until the mice were sacrificed on day 56.

### Cell culture

Embryonic stem cell-derived microglia (ESdM), a generous gift from Dr. Harald Neumann (University of Bonn Germany; Bonn, Germany), were cultured in DMEM/F12 50:50 (Thermo Fisher Scientific; Waltham, MA) containing N2 supplement (Invitrogen; Waltham, MA) with 0.048 mM l-glutamine (Thermo Fisher Scientific), 1 % d-glucose (Sigma-Aldrich; St. Louis, MO), and 1 % penicillin/streptomycin (Thermo Fisher Scientific). The ESdM are stable proliferating cells with most characteristics of primary microglia and are a suitable tool to study microglial function in vitro [17, 18].

### Antibodies and reagents

Reagents were purchased from the following sources: methamphetamine hydrochloride (METH) (Sigma-Aldrich); P2X7R antibody (Alomone; Jerusalem, Israel); tyrosine hydroxylase (TH) (Abcam; Cambridge, England); and pHrodo and Calcein-AM (Life Technologies; Waltham, MA). Poly-L-lysine (PLL), lipopolysaccharide (LPS), and cytochalasin D (Cyto D) were purchased from Sigma-Aldrich. Cell viability was determined by LIVE/DEAD assay (Invitrogen) and showed that METH at 1-1000 μM concentration had no toxic effects on microglia after 48 h of exposure (Additional file 1: Figure S1). The concentration of METH (100 μM) used in the present study is similar to other published studies [19, 20]. Optimal concentrations of cytochalasin D (5 μM), fractalkine (CX3CL1) (10 ng/mL), LPS (1 μg/ml), pHrodo (40 μg/ml), and Calcein (5 μM) were determined from dose- and time-dependent response studies.

### Immunohistochemistry

Brains of mice administered with escalating non-toxic doses of METH were harvested, fixed in 4 % paraformaldehyde, embedded in paraffin, and sectioned coronally at 5 μm. Following deparaffinization and dehydration, antigen retrieval was performed using Citrate Buffer reagent (Sigma-Aldrich). Sections were blocked with 4 % BSA and incubated with 1:100 dilution of antibodies specific for P2X7R (Alomone Labs) and Iba-1 (Wako) or 1:1000 TH (Abcam). Antibodies were detected using Alexafluor conjugates (1:500 for P2X7R and Iba-1 or 1:1500 for TH). DAPI was used for nuclear stain. The immunostained cells were observed using an Eclipse I-80 Microscope (Nikon, Melville, NY) fitted with a CoolSnap-EZ digital camera (Photometrics, Tucson, AZ). Image acquisition analysis was performed using NIS Elements R (Nikon) imaging software.

The expression of TH was quantified on ×20 images using ImageJ 1.42 software and analyzed based on integrated density, which is the intensity multiplied by the area of the particles analyzed (http://rsbweb.nih.gov/ij/) as described [21].

### RNA extraction and real-time qPCR

The change in the gene expression profile of the purinergic receptor P2X7 in ESdM cells was analyzed using real-time quantitative PCR (qPCR). ESdM cells were seeded at a density of 2 × 10^5^ cells per well in six-well plates followed by treatment with 100 μM METH for a time-course from 4 to 72 h (Additional file 2: Figure S2). Total RNA was isolated using the Quick-RNA™ MiniPrep as per manufacturer’s instructions (Zymo Research; Irvine, CA). RNA purity and concentration was determined using a NanoDrop ND-1000 spectrophotometer (Thermo Fisher Scientific). Conversion to complementary DNA (cDNA) was performed by reverse transcription using [1 μg of total RNA] the High-Capacity cDNA Reverse Transcription kit (Applied Biosystems; Foster City, CA). Primers were designed for P2X7R forward primer 5′-GAGGTGACGGAGAATGTC-3′, P2X7 reverse primer 5′-GCGCCTGGGATACTCAG-3′, and P2X7 probe 5′-/56-FAM/ACACTGCAGACTACACCTTCCC/36-TAMSp/-3′. cDNA (diluted 1:20) along with forward and reverse primers were mixed with Taqman Master mix. qPCR was performed on an Applied Biosystems StepOnePlus Real-Time PCR System (Applied Biosystems). The PCR conditions consisted of an initial melting cycle at 95 °C for 15 min, followed by 40 cycles of amplification at 95 °C for 15 s (denaturation), 60 °C for 30 s (annealing), and 72 °C for 30 s (extension). Raw data was analyzed using the ΔΔCt method (relative quantification). Results were expressed in relative gene expression levels (fold change) compared to the untreated control.

### Western blot analysis

To assess the change in protein expression of the receptor P2X7, ESdM cells (500,000 per T75 flask in 10-ml N2 medium) were incubated with 100 μM METH for 24, 48, and 72 h. IL-1β was used as a positive control. The cells were washed with PBS before protein was isolated for whole cell lysate using Cell Lytic Reagent and Protease Phosphatase Inhibitor Cocktail or protein fractionation using mammalian Protein Extraction Kit (Thermo Fisher Scientific). Cells were lysed per manufacturer’s instructions, and protein concentration was determined using the BCA assay method. Thirty micrograms of protein per lane was separated by SDS-gel electrophoresis using 10 % Tris-Glycine gels at 80 V. After gel electrophoresis, the whole cell lysate, membrane fraction, and cytoplasmic fraction proteins were transferred to nitrocellulose membranes overnight at 4 °C. Membranes with whole cell lysates and cytoplasmic fractions were blocked with 5 % milk before being immunostained overnight for rabbit anti-P2X7R (1:1000, Alomone; Jerusalem, Israel) or mouse anti-GAPDH (1:20,000, EMD Millipore MAB374; Darmstadt, Germany) as loading. Secondary HRP-linked polyclonal anti-rabbit or anti-mouse antibody (1:2000, Cell Signaling Technology; Danvers, MA) was incubated for 1 h at room temperature in 5 % milk before developing with SuperSignal West Femto Chemiluminescent Substrate (Thermo Fisher Scientific). Membranes with cell membrane fractions were first rinsed briefly in distilled water and stained with Ponceau S solution (Po-S) (0.5 [*w*/*v*] in 1 % [*v*/*v*] acetic acid) for 2 min, rinsed in distilled water to remove excess stain, and imaged using a G:Box Chemi HR16 (Syngene; Fredrick, MD) gel documentation system [22]. Protein levels were normalized to Ponceau-S used as loading control (70–100 kD) [20, 22]. Membranes were then blocked in 5 % milk for 1 h at room temperature followed by an overnight incubation with rabbit anti-P2X7R (1:1000, Alomone). Secondary HRP-linked polyclonal anti-rabbit (1:2000, Cell Signaling) was incubated for 1 h at room temperature in 5 % milk before developing with SuperSignal West Femto Chemiluminescent Substrate (Thermo Fisher Scientific). Membranes were imaged via G:Box Chemi HR16. Band intensities were measured, and normalized density values (OD) from the control were plotted.

### Phagocytosis assay

To assess phagocytosis, ESdM cells (50,000 cells per T25 flask in 5-ml N2 medium) were incubated with pH-sensitive pHrodo-conjugated *Escherichia coli* bioparticles (Life Technologies). Appropriate flasks were first treated for 48 h with either METH (100 μM) alone or with 1-h pretreatment of P2X7R antagonist A 438079 (10 μM). Cytochalasin D (5 μM) treated for 1 h was used as a negative control in separate flasks. Briefly, following treatment, cells were incubated for 1.5 h with pHrodo green bioparticles (40 μg/ml) at 37 °C in 5 % CO_2_. Immediately after incubation, cells were rinsed with cold phosphate buffered saline, scraped, and washed with FACS buffer (2 % BSA in PBS) before being re-suspended in 2 % paraformaldehyde and subjected to flow cytometric analysis by BD Canto II (BD Biosciences; Franklin Lake, NJ). Phagocytosis by microglia (FITC+) was quantified (to 10,000 events), and analysis was carried out using FACS DiVa software (Becton Dickinson) and FlowJo Software v 8.7 [23].

### Migration assay

Quantitative migration assays were carried out using 8-μm pore FluoroBlock migration plates (Calbiochem; Darmstadt, Germany) as described previously [23, 24]. ESdM cells were loaded with 5-mM Calcein-AM (Life Technologies) for 45 min at 37 °C and washed prior to seeding at 50,000 cells/well in the upper chamber of the tissue culture insert. CX3CL1 (10 ng/ml) was added to the lower chamber to stimulate migration. The number of migrated cells was counted using an inverted fluorescence live cell imaging system (Carl Zeiss MicroImaging; Thornwood, NY). Each experiment was performed in triplicate, and each experimental well was imaged five times in different locations, and the results were expressed as an average of the total number of migrated cells in response to chemoattractant under each experimental condition. The images were analyzed with AxioVision version 4.7 software (Carl Zeiss MicroImaging) and with National Institutes of Health ImageJ version 1.42 software (http://rsbweb.nih.gov/ij/) as described [25].

### siRNA knockdown of P2X7R

P2X7R expression in ESdM cells was silenced by small interfering RNA (siRNA) transfection for about 48 h. P2rx7 Trilencer-27 Mouse siRNA (OriGene Technologies; Rockville, MD) was used in conjunction with the jetPRIME transfection reagent (Polypus transfection™; Bioparc, France) to directly transfect the siRNA in N2 medium according to the transfection protocol. Briefly, ESdM cells were seeded at a density between 1-2 × 10^5^ in a T25 flask in N2 media. Five nanomolar P2X7R siRNA or scrambled siRNA was allowed to form duplexes with 8-μl jetPRIME transfection reagent in 400-μl jetPRIME buffer. Cells were transfected and incubated at 37 °C, and 5 % CO_2_ for 24 h before 2 ml of N2 media was added to each flask. Cells were incubated for up to 48 h and then tested for other functional readouts. Cells in medium alone served as controls. P2X7R-specific siRNA selectively knocked down the target as assessed by qPCR using GAPDH control.

### MSD proinflammatory cytokine panel

To further assess METH-induced changes in microglial function, we analyzed proinflammatory cytokine production using the Mouse ProInflammatory 7-Plex Tissue Culture Kit (MSD; Rockville MD) according to the manufacturer’s instructions. Briefly, P2X7R expression was silenced by siRNA transfection for about 48 h in ESdM cells, 1.5-2 × 10^5^ per T25 flask, as described above. Forty-eight hours after transfection, appropriate flasks were treated for 24, 48, or 72 h with METH (100 μM). Supernatants were collected, centrifuged to remove cellular debris, and concentrated using the Amicon Ultra-15 Centrifugal Filter Units (EMD Millipore; Darmstadt, Germany). Twenty-five microliters of undiluted supernatants was added to wells of the MSD plate in biological triplicates and incubated for 2 h with vigorous shaking at room temperature. Detection antibody solution was added to the wells, followed by further incubation for 1.5 h with vigorous shaking at room temperature. The plate was washed three times with PBS+ 0.05 % Tween-20, and 150 μl of 2X Read Buffer T was added to each well. Cytokine levels were estimated using provided standards and calculated by the SECTOR®Imager 2400A and MSD reader software (Meso Scale Discoveries, Rockville, MD, USA).

### Statistical analysis

Data were compared statistically using the one sample unpaired *t* test or a one-way analysis of variance (ANOVA) followed by post hoc Student Newman Keuls test to determine which conditions were significantly different from each other, and a Tukey posttest for multiple comparisons. Results were expressed as mean values (±SE), with values deemed statistically significant when *p* < 0.05.

## Results

### METH increased gene and protein expression of P2X7R in microglia

**Fig. 1:**
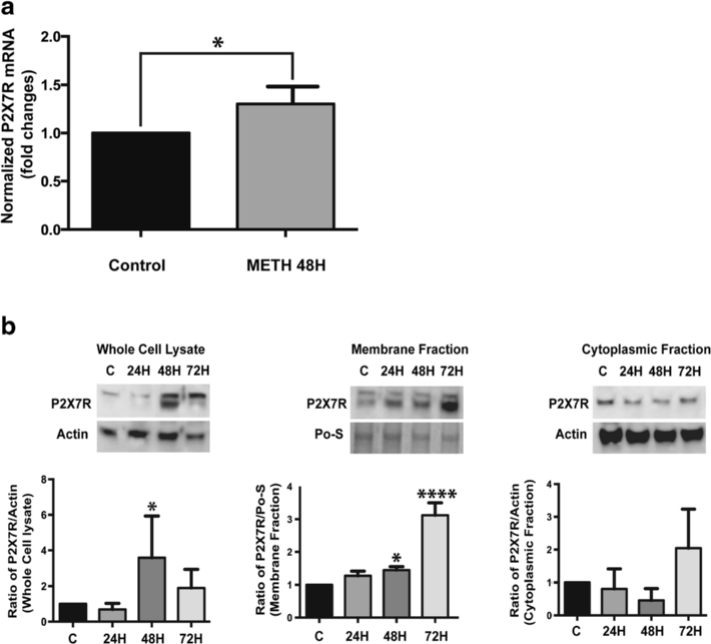
METH increases P2X7 purinergic receptor mRNA and protein expression in microglia. **a** The expression level of P2X7R mRNA in ESdM cells was modestly increased by treatment with 100 μM METH for 48 h (*P* < 0.0336). **b** Representative immunoblots of P2X7R in whole cell lysate, protein fraction, and Ponceau S staining (Po-S) as loading control. The ratios of P2X7R to protein loading control are shown in the histogram. *Error bars* represent mean ± SE of four independent experiments (*p* < 0.0003). (METH-treated versus control)

Several animal [26–28] and human studies [15, 29] have elucidated microglial involvement in METH neurotoxicity. Building evidence supports the notion that P2X7R are regulators of neuroinflammatory processes [30] and are upregulated on microglia [31, 32] in several different pathologies. To characterize P2X7R expression in the context of METH-induced microglia activation, we analyzed the gene and protein expression of P2X7R in the ESdM [18, 23] cells after 6, 24, 48, and 72 h of METH treatment (Additional file 2: Figure S2). Changes in the messenger RNA (mRNA) expression at the 48-h time point presented the most consistent and significant fold increase (*p* < 0.0336) (Fig. 1a).

Our analysis of P2X7R protein from total cell lysate by western blotting showed increased expression in a time-dependent manner after METH treatment (Fig. 1b, c). Interestingly, the P2X7R protein concentration in the cytoplasmic fraction (Fig. 1b, c) was reduced in comparison to the membrane fraction after METH treatment (48–72 h). These results are consistent with previously published reports that whole cell lysates and membrane fractions of the P2X7R protein (75 kD) also show a proteolytic product of the P2X7R around 60 kD [33]. These data suggest that not only is the P2X7R protein increased at 48 h and later time points but there may also be trafficking of P2X7 from the cytosol to the membrane.

### P2X7R antagonist reverses METH-induced microglial migration

**Fig. 2:**
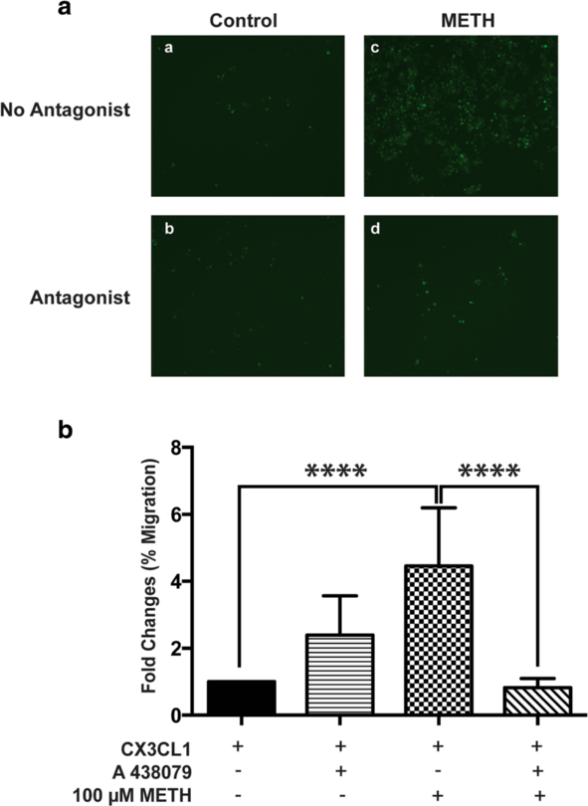
METH increases migratory capacity of microglia that is P2X7R-dependent. In vitro migration was performed using the transwell migration assay. ESdM cells (5 × 10^4^) were loaded with 5 mM Calcein-AM. In the lower chamber of the transwell plate, 10 ng/mL of fractalkine (CX3CL1) was added to determine the number of migrating cells and was visualized under ×40 magnification. **a** shows representative images of control (a), 10 μM P2X7R-antagonist A 438079 (b), 100 μM METH (c), and 100 μM METH and 10 μM P2X7R-antagonist A 438079 (d) treated ESdM cells. **b** Quantification of total cells migrated in response to 100 μM METH and/or A 438079 represents a statistically significant increase in migration towards CX3CL1 in METH-treated cells as compared to control (****p < 0.0001) that is reversed by pretreatment with A 438079 (****p < 0.0001) (ANOVA)

Microglial activation and subsequent migration are important to maintain CNS homeostasis in response to injury [34]. During the activation process, microglia switch from a ramified resting state to an amoeboid, motile phenotype capable of migration [34–36]. To determine the role of P2X7R in the effects of METH on microglial migration, ESdM cells were incubated with METH for 48 h and microglial migration was evaluated in response to the chemokine attractant, CX3CL1 using transwell migration assay [23] in the absence or presence of P2X7R competitive antagonist A 438079. As represented in Fig. 2, microglia migration was significantly (*p* < 0.0001) increased following METH treatment. This increase was significantly attenuated with a 1-h pretreatment with A 438079 (*p* < 0.0001) (Fig. 2) suggesting that P2X7R plays an important role in METH-induced modulation of microglial migration.

### Blockade of P2X7R exposure decreases METH-induced microglial phagocytosis

**Fig. 3:**
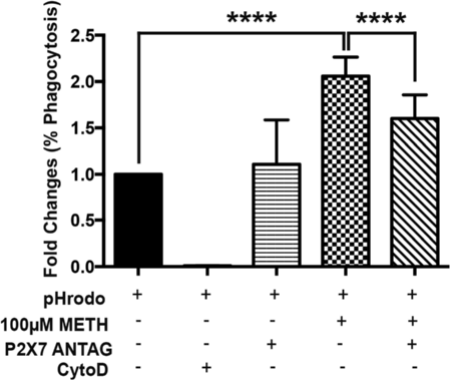
METH increases phagocytic capacity of microglia that is P2X7-dependent. Graphical representation of microglial phagocytosis in response to treatment with Cyto D (5 μM), A 438079 (10 μM), METH (100 μM), or METH (100 μM), and A 438079 (10 μM) was quantified using flow cytometry. METH (100 μM) significantly increases microglia phagocytosis (****p < 0.001). Pretreatment with antagonist decreases phagocytosis as compared to METH-treated cells. (****p < 0.0001) (ANOVA). Data consist of means ± SEM of three independent experiments

Microglial activation is characterized by a morphological change to an amoeboid phenotype capable of phagocytosis [34–36]. Since purinergic signaling is known to regulate microglial dynamics [37, 38] and facilitate microglial phagocytosis, we investigated the possibility that P2X7R plays a role in altered microglial phagocytic function following METH treatment. Microglial phagocytic activity was determined by quantifying fluorescence bright green *E. coli* bioparticle conjugates by flow cytometry [23]. The phagocytic activity represents the fold change of the percentage of the phagocytic cells in comparison with that of the control culture. Cells treated with Cyto D (5 μM), an inhibitor of cytoskeletal rearrangement, served as the experimental negative control. METH-treated microglia showed a statistically significant increase in phagocytosis (*p* < 0.0001) when compared to control. Pretreatment for 1 h with the P2X7R antagonist A 438079 prior to METH treatment prevented the METH-induced increase in phagocytosis compared to METH alone (*p* < 0.0001) (Fig. 3). These results suggest that P2X7R may play a role in modulating METH-induced microglial activation.

### P2X7R silencing decreases METH-induced microglial proinflammatory cytokine production

**Fig. 4:**
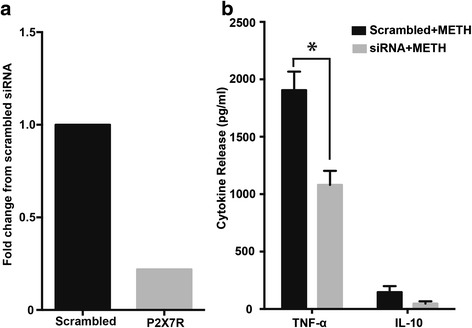
METH-induced TNF-α and IL-10 secretion is P2X7R dependent. P2X7R expression was silenced around 80% compared to scrambled siRNA in ESdM cells (**a**). Cytokines TNF-α and IL-10 showed trend toward increased expression after treatment with METH (100 μM) (data not shown). Following P2X7R silencing, there was a significant (*p < 0.0363) decrease in TNF-α in response to METH when compared to scrambled siRNA (**b**). Evaluation of IL-10 release showed increased trend with METH treatment followed by a decreased trend with P2X7R silencing, which was not statistically significant (**b**)

METH-induced microglial activation is known to promote the release of proinflammatory cytokines, such as IL-1ß and TNF-α [1, 39, 40]. To evaluate the role of METH in microglial activation, we analyzed markers of inflammation subsequent to P2X7R silencing (Fig. 4a). P2X7R gene expression was silenced for about 48 h continued with METH treatment for up to 48 h. Evaluation of proinflammatory cytokines in ESdM cells following METH treatment for 48 h showed increased production of TNF-a and IL-10 (data not shown). TNF-α expression was significantly (*p* < 0.0363) reduced in cells transfected with P2X7R siRNA when compared to cells transfected with scrambled siRNA (Fig. 4b). A similar trend of decreased IL-10 expression (Fig. 4b) in METH-treated ESdM cells was observed in P2X7R siRNA transfected cells when compared to cells transfected with scrambled siRNA P2X7R, suggesting that P2X7R regulates METH-induced production of these two cytokines.

### Chronic METH decreases tyrosine hydroxylase expression in vivo

**Fig. 5:**
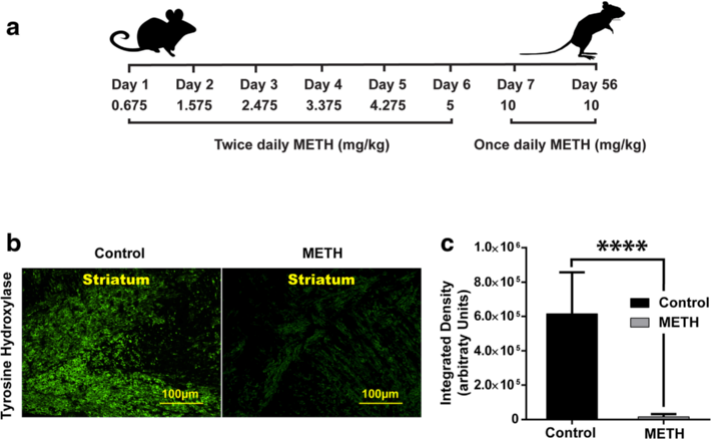
METH decreases tyrosine hydroxylase expression in vivo. Mice were subjected to an escalating METH dose schedule (s.c; twice daily) for 6 days, followed by a once daily administration of 10 mg/kg METH up to 56 days (**a**). Representative images of mouse coronal sections after 56 days are shown (**b**): Control (*n* = 3) and METH (*n* = 3) were stained for tyrosine hydroxylase. Five images at ×20 were taken for each mouse, and subsequent immunohistochemical analysis and quantification of tissue showed significant decreases (****p < 0.0001) in tyrosine hydroxylase expression in the striata of mice exposed to chronic METH treatment (**c**)

Most recreational METH abusers initially use lower doses, progressively increasing to higher doses and eventually engaging in increased amount and frequency of drug intake [41–43]. In this current study to simulate a similar pattern, we used an escalating METH dose schedule for our in vivo mouse experiments to investigate the deleterious effects of METH [41–44]. Male C57BL/6 mice were subjected to an escalating non-toxic dose of METH up to 10 mg/kg over a 7-day period (Fig. 5a), and immunohistochemical analysis of tyrosine hydroxylase (TH) was performed to evaluate dopaminergic depletion (Fig. 5b) [42, 45, 46]. In accordance with postmortem evaluation of its expression in humans and in vivo data from rodents, there was a dramatic depletion (*p* < 0.0001) (Fig. 5c) in TH within the striatum of METH-treated animals in comparison to control [47–49].

### Chronic METH increases P2X7R expression on murine microglia in vivo

**Fig. 6:**
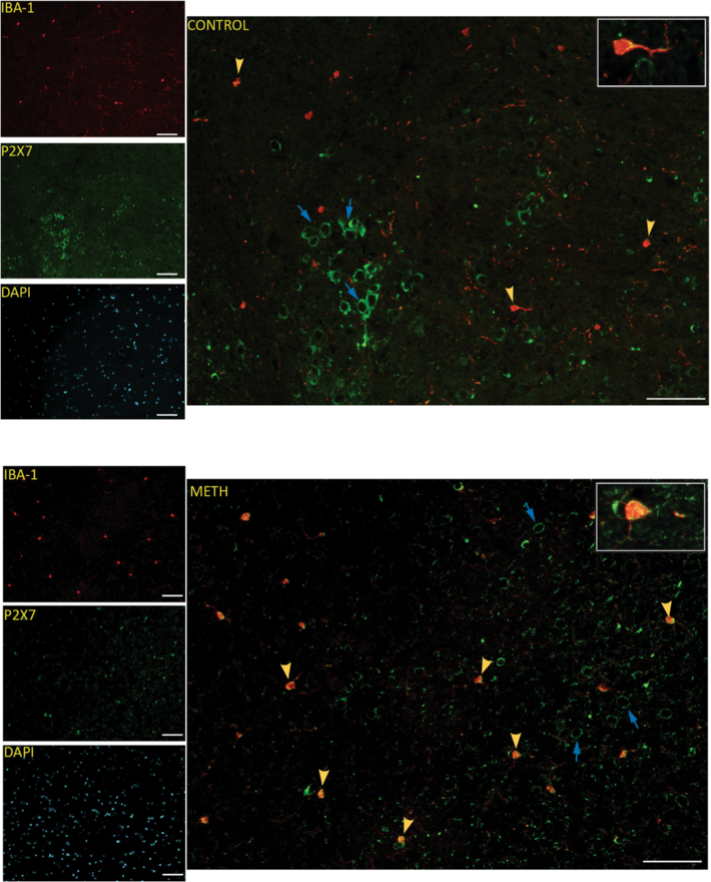
METH increases P2X7R expression in murine microglia in vivo. Representative images of immunohistochemical analysis of coronal sections from control and METH mice are shown: purinergic receptors (*green*), Iba-1 (*red*), and DAPI (*blue*). Images show the presence of P2X7R in neurons and an increase in P2X7R expression following escalating doses (to 10 mg/kg) of METH in microglia. *Yellow arrows* point to P2X7R co-staining in Iba-1-positive microglia, and *blue arrows* point to neuronal P2X7R staining. To highlight the co-staining, the larger images were taken under ×20 objective magnification (original scale bars, 50 μm) to show Iba-1 (*red*) and P2X7R (*green*) without DAPI. The *inset images* of co-staining in a single cell were taken under ×40 objective magnification. The *small images* on the left were taken under ×20 objective magnification (*original scale bars*, 50 μm)

To evaluate the association between the microglial P2X7R and METH-induced neuroinflammation, immunohistochemistry was performed for markers of purinergic receptor (P2X7R) and reactive microglia (Iba-1). In the control group, only a few microglia with thin and long processes were stained positive for P2X7R. However, a robust increase in Iba-1 immunoreactivity and hypertrophic cellular morphology of microglia was observed in the METH group that was particularly pronounced in the striatum in comparison to other regions of the brain. An increase in P2X7R immunoreactivity was observed in METH-treated mice compared to the saline group (Fig. 6). P2X7R expression (green) was observed in neurons and was increased concurrent with Iba-1 expression in microglia (Fig. 6). These results are consistent with reports of increases in P2X7R in patients suffering from neurodegenerative diseases and neuropathic pain [14]. This suggests that P2X7R may play a role in modulating METH-induced microglial activation.

## Discussion

In this study, we show that (1) METH upregulates microglia P2X7R mRNA and protein expression, (2) P2X7R antagonism inhibits METH-induced microglia migration and phagocytosis, and (3) P2X7R silencing decreases METH-induced cytokine production, especially of TNF-a, suggesting an important role of P2X7R in METH-induced cytokine regulation. To our knowledge, the study presented here provides evidence for the first time that P2X7R plays a role in mediating the effects of METH on microglia.

METH is a highly addictive psychoactive stimulant abused worldwide, and it is known to activate microglia [28]. The long half-life of METH of 12 h and duration of action contribute to METH abuse, neurotoxic oxidative stress, neuroinflammation, and excitatory neurotoxicity [12, 50]. Chronic METH abusers typically use doses between 5 to 1000 mg within a 24-h period, and studies suggest that even low, single doses of METH (0.10–1.00 mg/kg) are capable of inducing significant release of dopamine from the striatum of rhesus monkeys [51, 52]. Studies in rodents show that METH-induced chronic microgliosis precedes damage to dopaminergic terminals [15]. Furthermore, altered microglial function induces microglial proliferation 2 days after activation [6, 12, 53, 54].

P2X7R, a member of the P2X subfamily, is an ATP-sensitive ligand-gated ion channel permeable to calcium, sodium, and potassium ions upon activation [8, 55]. P2X7R is most highly expressed in macrophages/microglia [55, 56], but it is also expressed in neurons and astrocytes within the CNS [57–59]. The P2X7R is implicated in microglial activation; its expression is upregulated in a variety of neurodegenerative pathologies [2, 8]. Reports have shown that activation of P2X7 receptors during CNS pathology initiates the generation and release of mediators, which may contribute to the progression of neurotoxic pathologies [60–64]. Thus, P2X7R has been highlighted as a major contributor to microgliosis, which may lead to a deleterious cycle of neuroinflammation and subsequent neurodegeneration [64]. Therefore, we examined the role of P2X7R in METH-induced microgliosis and aberrant function, in vivo and in vitro by using stably proliferating ESdM cells that are morphologically and functionally similar to primary microglia [18, 65–67] and are an excellent tool to examine the role of purinergic receptors in microglia [23].

Expression of P2X7R mRNA (Fig. 1a) and protein (Fig. 1b, c) was increased upon METH treatments. Studies show that P2X7R expression is posttranscriptionally controlled in response to varying stimuli [2]. A recent report demonstrates that posttranslational modification of P2X7R by palmitic acid on carboxyterminal cysteines is required for its association with membranous lipid rafts [33]. P2X7R that are not palmitoylated are retained within the endoplasmic reticulum and are degraded by proteasomes [33]. Interestingly, in this study, we observed increased expression of P2X7R in the membranous fraction compared to the cytoplasmic fraction in ESdM exposed to METH (Fig. 1b, c). The significant increase in membranous P2X7R may be in part due to METH-induced release of the protein from cytoplasmic vesicular stores, perhaps by increased palmitoylation, in addition to translation of the increased P2X7R mRNA [33]. It is also plausible that P2X7R protein shuttles from the cytoplasm to the membrane subsequent to METH exposure. The involvement of P2X7R in vesicular trafficking is known [68]. Moreover, vesicular trafficking regulates the expression of another purinergic receptor isoform, P2X4R, which forms dimers with P2X7R [68, 69]. Taken together, this data suggests that a similar mechanism may control the expression of P2X7R.

Activation of P2X7R has been shown to induce microglial migration to the sites of injury and phagocytosis [12, 70]. Consistent with these reports, our studies show that P2X7R is an important regulator of microglial effector function in response to stimulant abuse. Engagement of P2X7R activates several secondary messengers and numerous intracellular signaling cascades downstream, thereby controlling trophic behaviors such as activation and proliferation [14, 64, 71–73]. In this study, we show that METH significantly increases microglial migration toward CX3CL1 compared to untreated control in a P2X7R-dependent manner (Fig. 2). CX3CL1, fractalkine, is a chemokine that is produced by neurons and is integral for maintaining neuron-microglia communication [74]. Extracellular CX3CL1 can influence a variety of microglial functions under physiological and pathological conditions, such as microglial synapse pruning during neuronal plasticity [74–76]. In the CNS, CX3CL1 acts as a chemoattractant and signals through the microglial CX3CR1 receptor to facilitate microglial migration along a concentration gradient [75]. Based on this phenomenon, we assessed microglial migration using a CX3CL1 gradient using a previously established protocol [23]. The increased migration of METH-treated microglia towards CX3CL1, which was blocked by pretreatment of the competitive P2X7R antagonist, is an indicator of METH-induced, P2X7R-dependent microglial activation (Fig. 2). Similarly, pharmacological inhibition of P2X7R reversed the METH-induced effect on microglial phagocytosis (Fig. 3).

METH-induced microgliosis involves the release of proinflammatory cytokines [77]. TNF-α is an important proinflammatory cytokine that can propagate neuroinflammation by initiating the production of other cytokines but may also act in a neuroprotective manner [77, 78]. In this study, we provide evidence of METH-induced P2X7R-dependent induction of TNF-α that may contribute to neuroinflammation (Fig. 4b). Microglial activation also has a role in neuroprotection, and the secretion of IL-10, an anti-inflammatory cytokine represents the dual role of this cell type within the CNS [79].

A goal of this study was to explore the role of purinergic receptors in METH-induced immunomodulatory responses in vivo. Route of administration [80] in addition to several other factors [81] determines the toxic effects of methamphetamine in animal models of chronic METH exposure. High-dose acute METH administration can lead to severe METH toxicity [82], and a major lethal effect of METH exposure in animal studies is development of hyperthermia. The escalating dose paradigm [45] in animal models is known to simulate the chronic METH abuser pattern while preventing the associated physiological consequences [41, 45, 46]. Importantly, evidence suggests that escalating dose models accurately mimic drug abuse patterns in humans [42, 45]. The chronic METH models demonstrate a decrease in dopamine concurrent with decreases in tyrosine hydroxylase similar to METH-induced neuropathological changes in humans [83–85]. Previous studies show that tyrosine hydroxylase, the rate-limiting enzyme in dopamine synthesis, is depleted with chronic METH administration [86]. Therefore, the METH-induced reduction of this important enzyme in dopaminergic neurons can contribute to decreased dopamine and has been used to estimate dopamine depletion and neuronal loss [48, 49, 76]. Using the escalating METH dose model, we found significant deficits in tyrosine hydroxylase immunoreactivity (Fig. 5), as reported in other chronic and acute binge models of METH [83]. The level of tyrosine hydroxylase in the brain tissue of the escalating METH dose mouse model was found to be correlated with lower dopamine levels, as estimated by HPLC in the brain tissue (unpublished studies). In addition, we show increased P2X7R expression in neurons as well as expression concurrent with Iba-1 staining for microgliosis in METH-treated mice (Fig. 6).

Numerous studies highlight the importance of P2X7R expression in microglial activation and neuroinflammation in several brain pathologies including multiple sclerosis and Alzheimer’s disease [64]. Chronic and long-term METH abuse damages multiple organ systems; however, none is more prominent than in the brain [27, 87]. Immunohistochemistry for reactive microglia (Iba-1) and P2X7R was particularly pronounced in the striatum of METH-treated animals. Interestingly, in METH abusers and children with prenatal METH exposure, neuroimaging studies demonstrate abnormalities in brain structure and chemistry, especially in the striatum [88]. Reactive microgliosis is observed in animal models of METH exposure and in the brains of human METH abusers in a process that is inversely correlated with the duration of METH abstinence [15]. Furthermore, MRS (magnetic resonance spectroscopy) studies performed in stimulant abuse patients (including METH and cocaine) to assess brain metabolites and neurochemicals in selected brain regions show higher concentrations in glia and correlate well with neuroinflammation [87].

Activation of purinoceptors has been indicated as a primary factor in microglial response [2]. A number of neurodegenerative conditions exhibit enhanced P2X7R expression in the neuroinflammatory foci where the presence of activated microglia is a concurrent feature [72, 73, 89, 90].

## Conclusions

Our findings in the present work converge in indicating that purinergic mechanisms contribute to altered microglia effector function due to METH. By investigating the pattern of P2X7R expression in vitro in ESdM microglia cells and in vivo in mice exposed to an escalating dose of METH, we show that microglia P2X7 receptors are upregulated in METH-induced microglia activation. This study sheds new light on the functional role of P2X7R in the regulation of microglial effector functions in the setting of substance abuse. P2X7R may thus play an important role in modulating neuroinflammatory responses by regulating pathologically activated glial cells in stimulant abuse. Antagonists for P2X7R could have potential therapeutic utility in the regulation of glial activation in stimulant abuse.

## Additional files

Additional file 1: Figure S1. ESdM cells were stained with Live/Dead Viability/Cytotoxicity assay to visualize cell death after 48 h treatment with METH. Cells did not display significant death in response to up to 1000 μM METH. (TIF 11093 kb) Additional file 2: Figure S2 The expression level of P2X7R mRNA in ESdM cells in response to 100 μM METH from 4 to 72 h was quantified using RT-PCR. (TIF 10526 kb)
