# Supplementary figures and images for: Methamphetamine alters microglial immune function through P2X7R signaling
Source: J Neuroinflammation. 2016 Apr 26;13:91. doi: 10.1186/s12974-016-0553-3 (PMC4847215; doi:10.1186/s12974-016-0553-3)

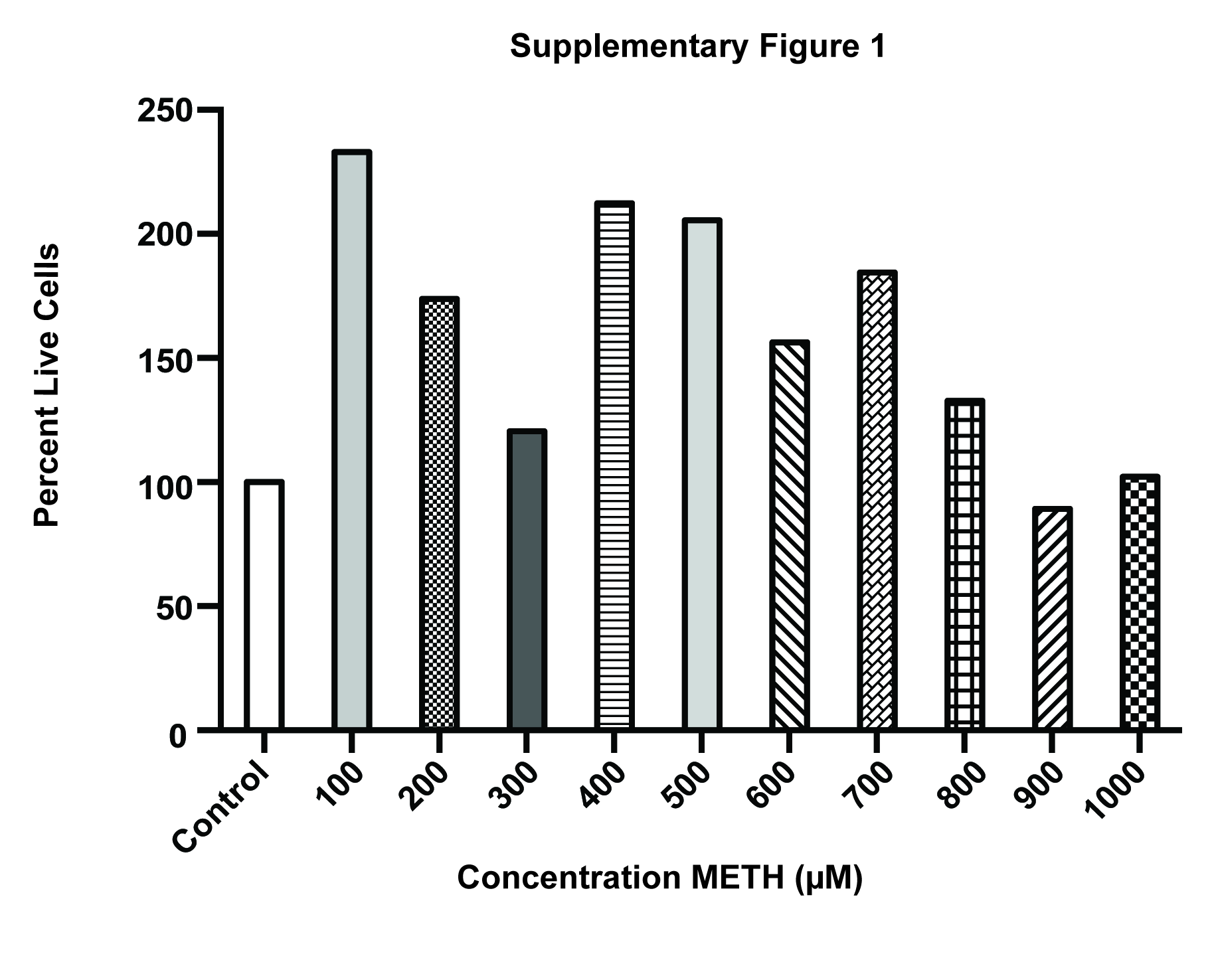

Supplement: Additional file 1: Figure S1. — ESdM cells were stained with Live/Dead Viability/Cytotoxicity assay to visualize cell death after 48 h treatment with METH. Cells did not display significant death in response to up to 1000 μM METH. (TIF 11093 kb) [file 12974_2016_553_MOESM1_ESM.tif]

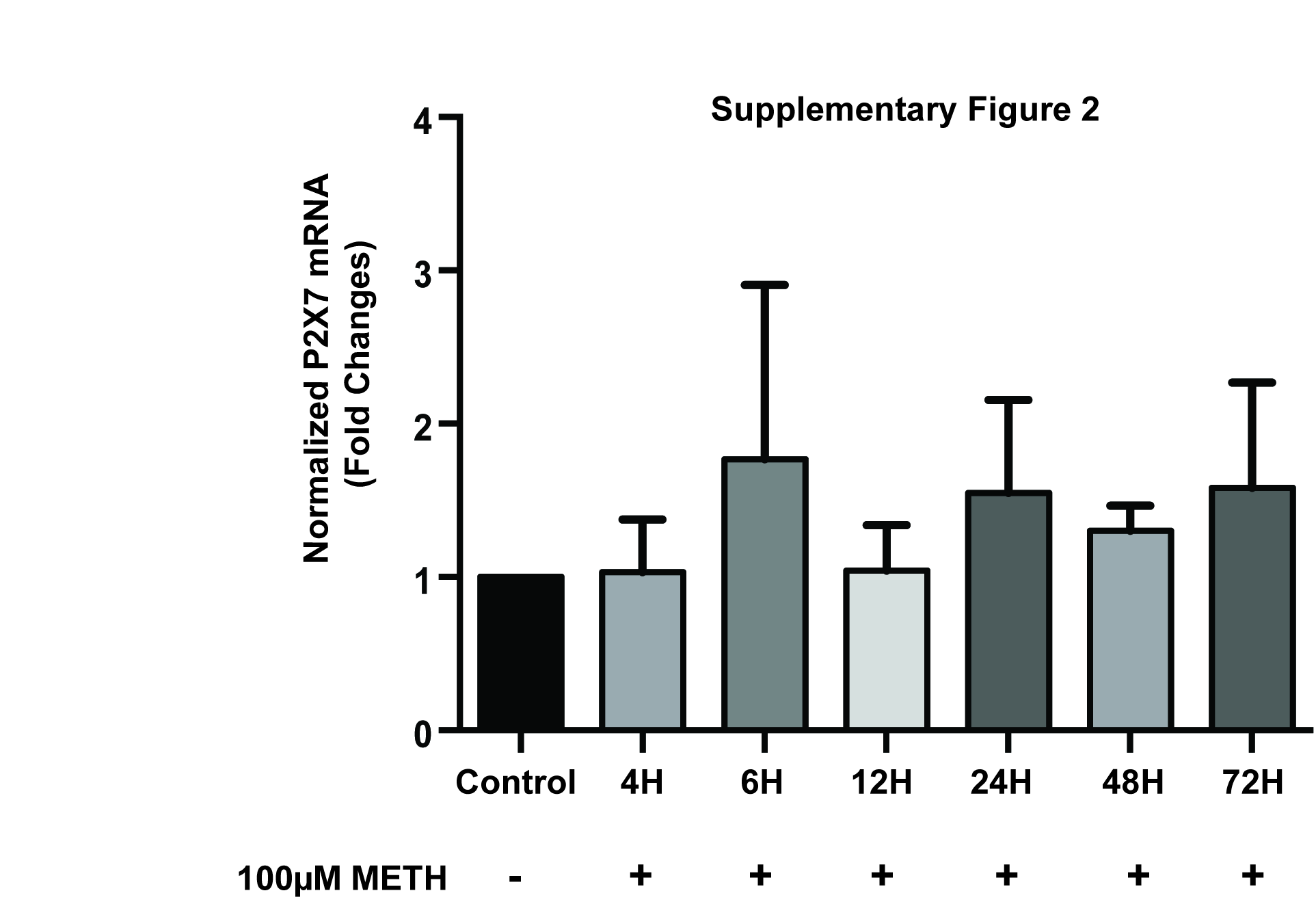

Supplement: Additional file 2: Figure S2 — The expression level of P2X7R mRNA in ESdM cells in response to 100 μM METH from 4 to 72 h was quantified using RT-PCR. (TIF 10526 kb) [file 12974_2016_553_MOESM2_ESM.tif]
